# Parliament and the Profession

**Published:** 1916-11-18

**Authors:** 


### Parliament and the Profession.

### An Irish Medical Officer.

THE Chief Secretary for Ireland informed Mr. Kilbride that the Local Government Board have declined to sanction the appointment of Dr. J. H. M'Kenna as temporary medical officer of the Monasterevan district as he is of military age and eligible for employment in the Army, there being urgent need for doctors in the R.A.M.C.; moreover, the Board is of opinion that a temporary arrangement made some time ago for the medical care of the district, which has a population of 4,600, is adequate and satisfactory.

### R.A M.C. Emoluments.

MR. FORSTER stated that there are two forms of commission in the R.A.M.C. Before the Military Service Act came into operation medical men joined the Army either under a special contract or through the Special Reserve. Since the Act came into force all medical men who have not enrolled with the Central Medical War Committee and are compelled to serve are taken into the Special Reserve. The pay of the Special Reserve is lower than that received under the contract, but when the gratuities given on cessation of employment are taken into consideration there is little difference in the emoluments.

### Nerve-Shattered Soldiers.

MR. HAYES FISHER informed Colonel Yate that the Statutory Committee are considering a scheme under which cases of uncertifiable nerve strain would be treated and trained in institutions where cases of ordinary wounds and illness other than mental are received. A further question put by Major Hunt to the Home Secretary elicited the information that the completion of the new Hampshire Asylum is being expedited for use as a military hospital, but that it is not intended to convert any other institutions under the Lunacy Authorities into military hospitals for the reception of uncertifiable cases of nerve strain, there being at the present time only two such institutions used for the cases in question, and these are entirely under the control of the War Office.

